# Aktualisierte Empfehlungen zur infektiologischen Versorgung von Flüchtlingen im Kindes- und Jugendalter in Deutschland (Stand 30. März 2022), angemeldet als S1-Leitlinie (AWMF-Register Nr. 048-017): Stellungnahme der Deutschen Gesellschaft für Pädiatrische Infektiologie e. V. (DGPI), der Gesellschaft für Tropenpädiatrie und Internationale Kindergesundheit e. V. (GTP), des Berufsverbandes der Kinder- und Jugendärzte e. V. (BVKJ) und der Deutschen Gesellschaft für Kinder- und Jugendmedizin e. V. (DGKJ), unter Mitarbeit des Berufsverbands der Ärztinnen und Ärzte des Öffentlichen Gesundheitsdienstes e. V. (BVÖGD), der Deutschen Gesellschaft für Kinderchirurgie e. V. (DGKCH), der Deutschen Gesellschaft für Gynäkologie und Geburtshilfe e. V. (DGGG), Deutsche Gesellschaft für Allgemeinmedizin und Familienmedizin (DEGAM), des Bündnis Kinder- und Jugendgesundheit e. V. (früher Deutsche Akademie für Kinder- und Jugendmedizin, DAKJ), der Deutschen Gesellschaft für Tropenmedizin, Reisemedizin und Globale Gesundheit e. V. (DTG), der Deutschen Gesellschaft für Globale und Tropenchirurgie e. V. (DTC) und der Pädiatrisch Infektiologischen Gruppe Schweiz (PIGS)

**DOI:** 10.1007/s00112-022-01499-4

**Published:** 2022-05-25

**Authors:** Johannes Pfeil, DGPI, Ralf Bialek, Ulrich Heininger, Johannes Liese, Arne Simon, August Stich, Kholoud Assaad, BVÖGD, Ulrich von Both, DAKJ/Bündnis Kinder- und Jugendgesundheit, Aleš Janda, Christa Kitz, Robin Kobbe, GTP, Mirjam Kunze, DGGG, Judith Lindert, DGKCH, Nicole Ritz, PIGS, Stefan Trapp, BVKJ, Roland Fressle, Markus Hufnagel, DGKJ

**Affiliations:** 1 Kinder- und Hausarztpraxis im Ärztehaus, Schnellerstr. 2, 74193 Schwaigern, Deutschland; 2 Gesundheitsamt Rhein-Neckar Kreis, Kurfürsten-Anlage 38–40, 69115 Heidelberg, Deutschland; 3 Abteilung für Pädiatrische Infektiologie, Dr. von Haunersches Kinderspital, Lindwurmstr. 4, 80337 München, Deutschland; 4 Universitätsklinik für Kinder- und Jugendmedizin, Eythstr. 24, 89075 Ulm, Deutschland; 5 Praxis für Kinder- und Jugendmedizin, Erwin-Vornberger-Platz 2, 97209 Veitshöchheim, Deutschland; 6 Zentrum für Innere Medizin, Institut für Infektionsforschung und Impfstoffentwicklung, STAKOB, Universitätsklinikum Hamburg-Eppendorf, Martinistr. 52, 20246 Hamburg, Deutschland; 7 Klinik für Frauenheilkunde, Universitätsklinikum Freiburg, Hugstetter Str. 55, 79106 Freiburg, Deutschland; 8 Klinik für Kinderchirurgie, Universitätsklinikum Schleswig-Holstein, Ratzeburger Allee 160, 23538 Lübeck, Deutschland; 9 Kinderspital, Luzerner Kantonsspital, Spitalstr., 6000 Luzern 16, Schweiz; 10 Praxis für Kinder- und Jugendmedizin, Huchtinger Heerstr. 26, 28259 Bremen, Deutschland; 11 Abteilung Pädiatrische Infektiologie und Rheumatologie, Klinik für Allgemeine Pädiatrie und Jugendmedizin, Zentrum für Kinder- und Jugendmedizin, Universitätsklinikum Freiburg, Mathildenstr. 1, 79106 Freiburg, Deutschland

**Keywords:** Flüchtling, Asylsuchende, Kinder, Infektiologische Versorgung, Empfehlungen, Refugee, Asylum seeker, Minor, Infectious disease, Recommendations

## Abstract

**Hintergrund:**

Mit etwa 190.000 Asylanträgen im Jahr 2021 ist Deutschland das wichtigste Aufnahmeland von Asylsuchenden in Europa.

Die vorliegenden Handlungsempfehlungen sollen eine Grundlage für eine evidenzbasierte und zielgerichtete infektiologische Versorgung minderjähriger Flüchtlinge schaffen.

**Ziele:**

Die Handlungsempfehlungen sollen medizinisches Personal in der Versorgung minderjähriger Flüchtlinge unterstützen, um

1. einen unvollständigen Impfschutz frühzeitig zu erkennen und zu vervollständigen;

2. übliche Infektionskrankheiten zu diagnostizieren und zu behandeln;

3. in Deutschland seltene Infektionskrankheiten frühzeitig zu erkennen und zu therapieren.

**Material und Methoden:**

Die Handlungsempfehlungen wurden als AWMF-Leitlinie Stufe 1 verfasst.

Entsprechend wurden die Empfehlungen durch eine repräsentativ zusammengesetzte Expertengruppe der beteiligten Fachgesellschaften im informellen Konsens erarbeitet und final von den Vorständen der Fachgesellschaften offiziell verabschiedet.

**Ergebnisse:**

Es werden Empfehlungen ausgesprochen, für den Umfang der Anamnese und der körperlichen Untersuchung minderjähriger Flüchtlinge. Für alle minderjährigen Flüchtlinge werden die Bestimmung eines Differenzialblutbildes sowie Untersuchungen auf Tuberkulose und Hepatitis B empfohlen.

Je nach Herkunft und Alter werden weitere gezielte Untersuchungen z. B. auf Hepatitis C, HIV oder Schistosomiasis empfohlen. Zur raschen Vervollständigung des Impfstatus wird eine alters- und indikationsbezogene Priorisierung einzelner Impfungen vorgenommen.

**Diskussion:**

Angesichts anhaltend hoher Flüchtlingszahlen ist eine weitere Professionalisierung der medizinischen Versorgung minderjähriger Flüchtlinge notwendig. Hierzu sollten die notwendigen strukturellen und personellen Rahmenbedingungen geschaffen werden.

## Abstract (en)

**Background:**

Based on 190,000 applications for asylum, Germany remains a top destination for refugees and asylum seekers in Europe. The updated recommendations are considered evidence-based and targeted guidelines for the diagnosis and prevention of infectious diseases in underage refugees and asylum seekers.

**Objective:**

The objective of these recommendations is to guide medical staff in the care of minor refugees, in particular to:

1. assure early recognition and completion of incomplete vaccination status,

2. diagnose and treat common infectious diseases,

3. recognize and treat imported infectious diseases that are considered uncommon to the German healthcare system.

**Material and methods:**

The recommendations have been formally written to be published as AWMF S1 guidelines.

This includes a representative expert panel appointed by several professional societies, and formal adoption of the recommendations by the board of directors of all societies concerned.

**Results:**

Recommendations are given for the medical evaluation of minor refugees, including medical history and physical examination. A blood count as well as screening for tuberculosis and hepatitis B should be offered to all minor refugees. In addition, screening for other infectious diseases like hepatitis C, HIV or schistosomiasis should be considered depending on age and country of origin. Vaccinations are recommended based on both age and country of origin.

**Conclusion:**

As thousands of minor refugees continue to seek shelter in Germany every year, professional health care with adequate financial support needs to be established to ensure an appropriate medical treatment of this particularly vulnerable population.

## Information

Diese Stellungnahme wurde von den beteiligten Autoren im Auftrag der jeweiligen Fachgesellschaften erarbeitet:

*Für die Deutsche Gesellschaft für Pädiatrische Infektiologie e.* *V. (DGPI):*

- PD Dr. med. Johannes Pfeil, Schwaigern
- PD Dr. med. Ulrich von Both, München
- Dr. Aleš Janda, MSc, PhD, Ulm
- PD Dr. med. Robin Kobbe, Hamburg
- Prof. Dr. med. Markus Hufnagel, Freiburg

*Für die Gesellschaft für Tropenpädiatrie und Internationale Kindergesundheit e.* *V. (GTP):*

- Dr. Christa Kitz, Veitshöchheim
- PD Dr. med. Robin Kobbe, Hamburg
- Dr. med. Judith Lindert, Lübeck

*Für den Berufsverband der Kinder- und Jugendärzte e.* *V. (BVKJ):*

- Dr. med. Stefan Trapp, Bremen

*Für die Deutsche Gesellschaft für Kinder- und Jugendmedizin e.* *V. (DGKJ):*

- Dr. Aleš Janda, MSc, PhD, Ulm
- Prof. Dr. med. Markus Hufnagel, Freiburg

*Für den Berufsverband der Ärztinnen und Ärzte des Öffentlichen Gesundheitsdienstes e.* *V. (BVÖGD):*

- Dr. med. Kholoud Assaad, Heidelberg

*Für die Deutsche Gesellschaft für Gynäkologie und Geburtshilfe e.* *V. (DGGG):*

- PD Dr. med. Mirjam Kunze, Freiburg

*Für die Deutsche Gesellschaft für Kinderchirurgie e.* *V. (DGKCH):*

- Dr. med. Judith Lindert, Lübeck

*Für das Bündnis Kinder- und Jugendgesundheit e.* *V. (früher Deutsche Akademie für Kinder- und Jugendmedizin, DAKJ):*

- PD Dr. med. Ulrich von Both, München

*Für die Deutsche Gesellschaft für Globale und Tropenchirurgie e.* *V. (DTC)*

- Dr. med. Judith Lindert, Lübeck

*Für die Pädiatrisch Infektiologische Gruppe Schweiz (PIGS):*

- PD Dr. med. Nicole Ritz, Luzern

*Mitarbeit*

An der Erarbeitung der Stellungnahme waren außerdem beteiligt:

- Für die *Deutsche Gesellschaft für Pädiatrische Infektiologie e. V. (DGPI)*:
  - Prof. Dr. med. Ralf Bialek
  - Prof. Dr. med. Ulrich Heininger
  - Prof. Dr. med. Johannes Liese
  - Prof. Dr. med. Arne Simon
- Für den *Berufsverband der Kinder- und Jugendärzte e. V. (BVKJ)*:
  - Dr. med. Roland Fressle
- Für das *Bündnis Kinder- und Jugendgesundheit e. V.* (früher *Deutsche Akademie für Kinder- und Jugendmedizin, DAKJ; Sprecher der Kommission für Infektionskrankheiten und Impffragen)*
  - Prof. Dr. med. Ulrich Heininger
- Für die *Deutsche Gesellschaft für Kinder- und Jugendmedizin e. V. (DGKJ)*:
  - Prof. Dr. med. Johannes Liese
- Für die *Deutsche Gesellschaft für Tropenmedizin, Reisemedizin und Globale Gesundheit e. V. (DTG)*
  - Prof. Dr. med. August Stich

## Einleitung

Bürgerkriege, Naturkatastrophen und Armut führten in den Jahren 2015 und 2016 mehr als eine Million Menschen als Flüchtlinge nach Deutschland. Dadurch ergaben sich erhebliche gesellschaftliche Herausforderungen, die weitreichende und bis heute anhaltende Veränderungen nicht nur in Deutschland, sondern in ganz Europa auslösten.

Auch in den letzten Jahren bleibt die Zahl flüchtender Menschen hoch. Weltweit sind nach Angaben des UNHCR im Jahr 2022 über 80 Mio. Menschen auf der Flucht. Mit zuletzt gut 190.000 neuen Asylanträgen im Jahr 2021, davon etwa 73.000 (entsprechend 39 %) von Minderjährigen, bleibt Deutschland eines der Hauptaufnahmeländer von Asylsuchenden in Europa [1]. Angesichts des Krieges in der Ukraine, anhaltender weltweiter Konflikte und den immer bedrohlicher werdenden Auswirkungen des weltweiten Klimawandels ist davon auszugehen, dass auch in den kommenden Jahren sehr viele Menschen weltweit Schutz in Ländern außerhalb ihrer Heimat suchen werden.

Flüchtlinge und Asylsuchende sind eine besonders vulnerable Gruppe in unserer Gesellschaft. Aus medizinischer Sicht besteht eine Gefährdung durch die möglicherweise mangelhafte medizinische Versorgung im Herkunftsland und während der Flucht, bei gleichzeitig besonderer gesundheitlicher Gefährdung. Nach der Ankunft in Deutschland erschweren sprachliche, soziale und teilweise auch ökonomische Barrieren die gesundheitliche Versorgung. Flüchtlinge benötigen daher angemessene und niederschwellige medizinische Angebote, die an die individuelle Situation angepasst sein müssen. Es ist eine professionelle, soziale und ethische Herausforderung, die medizinische Versorgung von Flüchtlingen adäquat zu organisieren und durchzuführen. Dies gilt insbesondere für Kinder und Jugendliche, die teilweise als unbegleitete minderjährige Flüchtlinge (UMF) nach Deutschland kommen.

Wesentliche Voraussetzungen für eine tragfähige medizinische Versorgung von Flüchtlingen im Kindes- und Jugendalter sind im Bremer Modell dargestellt [2]. Hierin wird das Ziel formuliert, ein sinnvolles medizinisches Angebot für Flüchtlinge bereitzustellen. Eine von den Autoren ausdrücklich befürwortete Grundhaltung zur ärztlichen Versorgung von minderjährigen Flüchtlingen leitet sich unabhängig von ihrem Rechtsstatus aus dem Artikel 24 [Gesundheitsvorsorge] der *UN-Kinderrechtskonvention *aus dem Jahr 1989 her. Die Vertragsstaaten erkennen hierin das Recht des Kindes auf das erreichbare Höchstmaß an Gesundheit an. Minderjährige Flüchtlinge sind auf dem gleichen medizinischen Niveau zu versorgen wie die einheimische Bevölkerung.

Der deutsche wie auch der europäische Gesundheitssektor benötigen hierfür geschultes medizinisches Personal, welches in der Lage ist, die Gesundheitsrisiken und -bedürfnisse dieser Kinder und Jugendlichen zu erkennen und eine kompetente Betreuung zu gewährleisten.

Um das Ziel eines umfassenden medizinischen Angebotes für Flüchtlinge zu erreichen, ist es notwendig, den medizinischen Bedarf von minderjährigen Flüchtlingen und ihrer Familien zu kennen. In einem Interview mit Flüchtlingen in Schweden zeigte sich, dass ein routinemäßig durchgeführtes infektiologisches Screening überwiegend als unerfreuliche Pflichtuntersuchung angesehen wurde. Bemerkenswert ist hierbei vor allem, dass die Mehrheit der Flüchtlinge angab, nicht über Art und Umfang der durchgeführten Screeninguntersuchungen informiert worden zu sein [3].

Wichtige infektiologische Fragestellungen bei der Betreuung von minderjährigen Flüchtlingen sind insbesondere die Sicherstellung eines vollständigen Impfschutzes, aber auch die Diagnostik und Therapie von teils importierten und seltenen Infektionskrankheiten trotz Sprachbarrieren, Sammelunterkünften und unterschiedlichem kulturellen Hintergrund.

In einer gemeinsamen Stellungnahme hatten die Deutsche Gesellschaft für Pädiatrische Infektiologie (DGPI), die Gesellschaft für Tropenpädiatrie und Internationale Kindergesundheit (GTP) und der Berufsverband der Kinder- und Jugendärzte (BVKJ) erstmals im Jahr 2015 Empfehlungen für die infektiologische Versorgung von Flüchtlingen im Kindes- und Jugendalter veröffentlicht [4]. Seither konnten die Mitarbeiter im Gesundheitssystem in vielen Bereichen neue Erfahrungen in der medizinischen Versorgung von Flüchtlingen gewinnen, und zahlreiche Untersuchungen ergaben neue Erkenntnisse bezüglich des medizinischen Bedarfs sowie der Prävalenz von spezifischen Infektionserkrankungen bei Flüchtlingen unterschiedlicher Herkunft. Ebenso wurden seit dem Jahr 2015 mehrere nationale Empfehlungen zur infektiologischen Untersuchung von Flüchtlingen im Kindes- und Jugendalter veröffentlicht [5].

Die vorliegende Aktualisierung der Empfehlungen zur infektiologischen Versorgung von Flüchtlingen im Kindes- und Jugendalter in Deutschland berücksichtigt diese neuen Erkenntnisse, die nach Sichtung der Literatur zusammengefasst wurden.

Die vorliegenden Empfehlungen sollen Ärzte^1^/medizinisches Personal in der medizinischen Versorgung von Flüchtlingen im Kindes- und Jugendalter unterstützen, mit dem Ziel,

1. einen unvollständigen Impfschutz frühzeitig zu erkennen und rasch zu vervollständigen – zum individuellen Schutz und um Ausbreitungen von Infektionskrankheiten zu verhindern;
2. übliche Infektionskrankheiten im Kindes- und Jugendalter, auch vor dem Hintergrund von Sammelunterkünften, Sprachbarrieren und unterschiedlichen kulturellen Auffassungen, zu diagnostizieren und zu behandeln;
3. in Deutschland seltene Infektionskrankheiten (z. B. Tuberkulose, Malaria, Schistosomiasis, kutane Leishmaniose) frühzeitig zu erkennen und zu therapieren.

Um die Stellungnahme für den Alltag anwendbar zu machen, wurden in der vorliegenden Aktualisierung die konkreten Handlungsempfehlungen möglichst knapp zusammengefasst. Eine ausführliche Begründung für die Handlungsempfehlungen mit der zugrunde liegenden Literatur findet sich im Abschn. „Begründung der vorgeschlagenen Screeninguntersuchungen“. Die vorliegende Empfehlung konzentriert sich bewusst auf die Infektionsdiagnostik und Infektionsprävention. Dabei ist es selbstverständlich, dass Kindergesundheit weit über diese infektiologischen Gesichtspunkte hinaus reicht und insbesondere auch psychische Gesundheit, Integration und Bildungschancen wesentliche Aspekte für ein gesundes Aufwachsen von Flüchtlingen im Kindes- und Jugendalter darstellen.

## Allgemeine Untersuchung bei Ankunft und im Verlauf

### Allgemeine Aspekte

Seit 2015 zeigte sich sehr klar, dass die meisten ambulanten und stationären Behandlungen bei Flüchtlingen im Kindes- und Jugendalter aufgrund von banalen, auch bei uns üblichen Infektionserkrankungen erfolgen. Am häufigsten sind hierbei respiratorische Infektionen, gefolgt von Hauterkrankungen und gastrointestinalen Infektionen [6–8].

Für die Herkunftsländer spezifische und in Deutschland seltene Infektionskrankheiten wie HIV-Infektion, Hepatitis A, B, C und E, Tuberkulose oder Malaria finden sich insgesamt nur bei einem kleinen Teil der Flüchtlinge. Für die Prävalenz dieser Erkrankungen und damit einhergehende Empfehlungen für infektiologische Untersuchungen sind generell die Herkunft sowie das Alter der untersuchten Personen entscheidend [9–11]. Neben Infektionskrankheiten gibt es andere Erkrankungen und Gesundheitsprobleme, die gehäuft bei minderjährigen Flüchtlingen auftreten und dadurch auch in der Untersuchung besondere Beachtung erfahren sollten. Beispiele sind die sehr häufig feststellbaren Zahnerkrankungen [8] sowie Anämien [12]. Insbesondere bei UMF treten gehäuft psychische Erkrankungen auf [13, 14].

Problematisch sind im Alltag häufig sprachliche und kulturelle Barrieren. Wesentliche Rahmenbedingungen für eine gelingende Untersuchung sind in Tab. 1 zusammengefasst. So selbstverständlich diese Aspekte zunächst erscheinen, so schwierig ist es doch diese im medizinischen Alltag konsequent umzusetzen. Insbesondere die Sprachbarriere ist ein häufiges Hindernis für eine gelingende medizinische Versorgung [15]. Sinnvoll ist es daher, wann immer möglich, die Untersuchungen im Voraus zu planen und mit Hilfe von, aus der Sicht des behandelnden Arztes, vertrauenswürdigen Dolmetschern durchzuführen. Je nach Untersuchungsort können gegebenenfalls auch fremdsprachiges Personal, Videodolmetscher oder spezielle Anamnesebogen eine ausreichende Kommunikation ermöglichen.

**Table 1:** 

Flüchtlinge im Kindes- und Jugendalter sollten während medizinischer Untersuchungen, wann immer möglich, von mindestens einem Elternteil oder Sorgeberechtigten begleitet sein
Mit den Flüchtlingen bzw. derer Begleitperson(en) sollte eine ausreichende Kommunikation möglich sein
Im Rahmen der Untersuchung sollte aktiv nach Gesundheitsproblemen gefragt werden

### Anamnese

Tab. 2 gibt einen Überblick über wesentliche Punkte der Anamnese. Nicht in jeder Untersuchungssituation wird eine vollständige Anamnese möglich und hilfreich sein. Allerdings sollte, soweit möglich, aktiv nach Gesundheitsproblemen und Vorerkrankungen gefragt werden. Insbesondere für die dauerhafte Betreuung sollte die Anamnese auch die Erhebung der Familienanamnese sowie der Anamnese zur Flucht umfassen.

**Table 2:** 

*A) Aktuelle Beschwerden und Vorerkrankungen?*
Bestehen aktuelle Gesundheitsprobleme?
Gibt es Hinweise auf übertragbare Erkrankungen? (Z. B. exanthematische Hauterkrankungen, Durchfall, Atemwegserkrankungen, inkl. Tuberkulose? Enger Kontakt zu Infizierten?)
Bestehen chronische Erkrankungen?
Stattgehabte Operationen?
Wie ist der Impfstatus?
Werden Medikamente eingenommen?
Wie ist die Ernährung (insbesondere bei Säuglingen und Kleinkindern)?
Sind Allergien bekannt?
*B) Familienanamnese und Begleitpersonen*
Woher stammt das Kind/die Familie?
Welche Familienangehörigen begleiten das Kind?
Wie ist die Wohnsituation?
Beruf und berufliche Situation der Eltern
Gibt es weitere Sorgeberechtigte/Kontaktpersonen?
Liegen die Telefonnummern/Kontaktdaten der Familienangehörigen und ggf. weiterer Kontaktpersonen vor?
Wie ist das Kind in Deutschland betreut (Kindergarten, Schule, Ausbildung etc.)?
Gibt es besondere familiäre Belastungen/Erkrankungen?
Gibt es übertragbare Krankheiten in der Familie (insbesondere Tuberkulose)?
*C) Anamnese zur Flucht*
Dauer der Flucht
Fluchtroute/Aufenthalte während der Flucht
Bedingungen der Flucht (medizinische Versorgung, traditionelle Prozeduren, Begleitung, ggf. Schulbesuch)
Traumatisierende Erfahrungen, Gewalterfahrung (inkl. sexuelle Gewalt)
Unfälle

### Klinische Untersuchung

Tab. 3 gibt einen Überblick über wesentliche Bestandteile der klinischen Untersuchung. Auch hier wird der Umfang der Untersuchung je nach Behandlungsanlass variieren. Um mögliche Auffälligkeiten frühzeitig entdecken und ggf. behandeln zu können, sollten die in der Tabelle aufgeführten Untersuchungen aber möglichst rasch nach Ankunft in Deutschland erfolgen.

**Table 3:** 

*A) Anthropometrische Daten*
Länge, Gewicht (inkl. Gewichtsverlauf), BMI, Kopfumfang (mit Eintragung in Perzentilenkurven [16])
*B) Allgemeine körperliche Untersuchung*
Haut (z. B. Infektionen, inkl. Skabies, akute Verletzungen, Narben)
Herz, Lunge, Abdomen
Skelettsystem (Hüfte, Gangbild, Rachitis-Zeichen)
Lymphknotenstatus
HNO und Zahnstatus (Karies?)
Orientierende neurologische Untersuchung
Orientierender psychischer Status
Genitale (Pubertätsstadium, Position Hoden, Hinweise auf Infektionen, Missbrauch oder weibliche Genitalverstümmelung („female genital mutilation“, FGM))
Blutdruck
*C) Weitere Untersuchungen*
Altersentsprechende Sehprüfung (z. B. Lang-Stereotest, Lea-Test)
Hörtest
Prüfung des Entwicklungsstatus

Der folgende Abschnitt gibt einen kurzgefassten Überblick über empfohlene Untersuchungen bei Flüchtlingen im Kindes- und Jugendalter. Hierbei sind insbesondere Herkunft und Alter der Flüchtlinge sowie Dauer und Art der Flucht zu berücksichtigen. Detaillierte Informationen und Literaturangaben zu den empfohlenen Untersuchungen sind im Abschn. „Begründung der vorgeschlagenen Screeninguntersuchungen“ aufgeführt. Grundsätzlich muss sichergestellt werden, dass positive Screeningbefunde nicht nur übermittelt werden, sondern in eine adäquate Abklärung und ggf. Therapie münden.

### Generell empfohlene Laboruntersuchungen

#### Blutbild

Nach Ankunft in Deutschland sollte ein Differenzialblutbild bestimmt werden.

Hierdurch können relevante Anämien entdeckt werden und nachfolgend eine Behandlung (Eisenmangelanämie) bzw. auch weitere Abklärung bei Verdacht auf hereditäre Hämoglobinopathien (insbesondere Thalassämie und Sichelzellerkrankung) eingeleitet werden.

#### Screening auf Tuberkulose

Ein Screening auf Tuberkulose soll frühzeitig nach der Ankunft bei allen minderjährigen Flüchtlingen, insbesondere bei Kindern < 5 Jahren, erfolgen. Sofern das Screening nicht bereits in der Erstaufnahmeeinrichtung erfolgt ist, soll es im Verlauf durch andere Stellen wie z. B. Kinder- und Jugendärzte sowie Allgemeinärzte durchgeführt und dokumentiert werden und damit ein fester Bestandteil der Versorgung von Kindern und Jugendlichen mit Migrations- bzw. Fluchthintergrund sein.

Allen geflüchteten Kindern und Jugendlichen, insbesondere jungen Kindern (< 5 Jahre) mit einer latenten tuberkulösen Infektion (LTBI), soll eine Chemoprävention angeboten werden.

Das Screening wird im Kindes- und Jugendalter bevorzugt mittels Interferon-gamma Release Assay (IGRA) durchgeführt. Bei Kindern unter 5 Jahren kann alternativ ein Tuberkulin-Hauttest (THT) angelegt werden. Bei Jugendlichen ab dem 15. Geburtstag, die in eine Gemeinschaftsunterkunft für Flüchtlinge oder Asylsuchende aufgenommen werden, ist nach der Novellierung des Infektionsschutzgesetzes vom Januar 2019 (§ 36 Absatz 4 IfSG) eine Röntgenuntersuchung der Lunge nicht mehr zwangsläufig vorgeschrieben und kann stattdessen durch einen immunologischen Test (bevorzugt IGRA) ersetzt werden. Im Fall eines positiven immunologischen Testergebnisses bzw. bei anamnestischen oder klinischen Hinweisen auf das Vorliegen einer Tuberkulose soll eine Thorax-Röntgenuntersuchung durchgeführt werden.

Eine routinemäßige Röntgenuntersuchung des Thorax zum Ausschluss einer Lungentuberkulose ist bei Kindern und Jugendlichen unter 15 Jahren aufgrund der niedrigen Sensitivität bei relevanter Strahlenbelastung jedoch abzulehnen.

Die Interpretation des IGRA- bzw. THT-Ergebnisses muss unter Berücksichtigung des BCG-Impfstatus und des Ernährung- bzw. Krankheitsstatus (falsch-negativ z. B. bei Immunsuppression und schwerer Malnutrition) erfolgen. So ist beispielsweise in der Ukraine die BCG-Impfung eine Standardimpfung. Bei Durchführung des THT muss nach 48 bis 72 h eine mögliche Induration abgelesen werden. Generell gilt eine Induration von 5 mm oder größer als positiv und sollte weiter abgeklärt werden. Bei einer Induration von 10 mm oder größer ist ein falsch-positives Testergebnis auch bei vorangegangener BCG-Impfung sehr unwahrscheinlich.

Detaillierte Informationen zu Diagnostik und Therapie der Tuberkulose finden sich in der AWMF Leitlinie „Diagnostik, Prävention und Therapie der Tuberkulose im Kindes- und Jugendalter“ [17].

#### Hepatitis B

Zur Abklärung einer Hepatitis-B-Virus(HBV)-Infektion sollte allen minderjährigen Flüchtlingen und Schwangeren eine Bestimmung des HBs-Antigens angeboten werden.

Hierdurch werden chronische HBV-Infektionen in den meisten Fällen (cave: falsch-negative Ergebnisse bei „Escape“-Mutationen) zuverlässig erkannt. Dies ermöglicht eine weitere Abklärung und gegebenenfalls Therapie infizierter Personen entsprechend der aktuellen AWMF-Leitlinie zu Prophylaxe, Diagnostik und Therapie der Hepatitis-B-Virus-Infektion [18].

### Herkunfts- und indikationsabhängig empfohlene Laboruntersuchungen

#### Hepatitis C

Zur Abklärung einer Hepatitis-C-Virus(HCV)-Infektion sollte insbesondere minderjährigen Flüchtlingen aus Hochprävalenzgebieten (Afrika, Naher und Mittlerer Osten, Osteuropa) eine Bestimmung von Anti-HCV-Antikörpern angeboten werden. Hierdurch wird eine chronische HCV-Infektion zuverlässig erkannt und die weitere Überwachung und Therapie infizierter Personen ermöglicht.

#### HIV

Zur Abklärung einer HIV-Infektion sollte insbesondere minderjährigen Flüchtlingen aus Hochprävalenzgebieten (Subsahara-Afrika, Osteuropa) ein HIV1/2-Suchtest (HIV-p24-Antigen + HIV1/2-Antikörper) angeboten werden. Zudem sollen alle schwangeren Frauen im Rahmen der generellen Infektionsdiagnostik eine HIV-Diagnostik erhalten, um eine rechtzeitige antiretrovirale Therapie zu gewährleisten und eine vertikale HIV-Übertragung wirksam zu verhindern. Bei anamnestisch bestehendem Verdacht auf eine kürzlich stattgehabte Infektion (ebenso bei Hepatitis B und C) ist ggf. eine PCR-Diagnostik angezeigt.

#### Schistosomiasis/Bilharziose

Bei Flüchtlingen mit Herkunft aus Endemiegebieten der Schistosomiasis (insbesondere Subsahara-Afrika und Gebiete entlang des Nils) ist eine Schistosomiasis-Serologie empfohlen.

Bei positiver Serologie werden parasitologische Stuhl- (3 Stuhlproben) und Urinuntersuchungen (bis zu 3‑mal mittäglicher Sammelurin zwischen 10 und 14 Uhr) durchgeführt und eine Behandlung bei Nachweis von Schistosomen-Eiern mit Praziquantel eingeleitet [19]. Bei den Stuhl- und Urinuntersuchungen ist die lange Präpatenzzeit von bis zu drei Monaten zu beachten. Dies bedeutet, dass die serologische Diagnostik, aber vor allem die parasitologischen Untersuchungen erst drei Monate nach der letzten Süßwasserexposition im Endemiegebiet zuverlässige Ergebnisse liefern. Bei Verdacht auf eine akute Bilharziose (Katayama-Fieber) ist eine PCR-Diagnostik aus EDTA-Blut angezeigt [19].

#### Strongyloidose

Insbesondere vor einer geplanten Immunsuppression sowie bei Eosinophilie ist bei Flüchtlingen aus Afrika, Asien, Mittel‑, Südamerika und Ozeanien eine Untersuchung auf Strongyloidose empfohlen.

Diese erfolgt initial durch eine serologische Untersuchung. Positive Serologien werden durch Stuhluntersuchungen (3 Stuhlproben, inkl. Baermann-Test) weiter abgeklärt und ggf. mit Ivermectin behandelt.

#### Chagas-Krankheit (*Trypanosoma cruzi*)

Bei minderjährigen Flüchtlingen, insbesondere bei jungen Frauen im gebärfähigen Alter und Schwangeren sowie Blut- und Organspendern aus Endemiegebieten Mittel- und Südamerikas (Argentinien, Belize, Bolivien, Brasilien, Chile, Costa Rica, Ecuador, El Salvador, Französisch-Guayana, Guatemala, Guayana, Honduras, Kolumbien, Mexico, Nicaragua, Panama, Paraguay, Peru, Surinam, Uruguay und Venezuela) ist eine serologische Untersuchung auf *Trypanosoma cruzi* empfohlen (eine Leitlinie zur Diagnostik und Therapie der Chagas-Erkrankung wird in Kürze auf der AWMF-Webseite publiziert werden).

### Nichtempfohlene Laboruntersuchungen

#### Stuhluntersuchungen als Screening auf Darmparasiten

Ein generelles Screening aller Flüchtlinge im Kindes- und Jugendalter auf Darmparasiten ist nicht empfohlen. Die hierfür notwendigen mehrfachen Stuhluntersuchungen sind in der Praxis üblicherweise nicht praktikabel und/oder weisen eine zu niedrige Sensitivität auf [20].

Bei klinischem Verdacht auf eine chronische intestinale Infektion (z. B. chronische Diarrhö, Anorexie, Gedeihstörung, Meteorismus, Anämie und/oder Eosinophilie) sollte eine gezielte Untersuchung auf Darmparasiten erwogen werden. Bei positiven Befunden erfolgt eine zielgerichtete Therapie.

Eine präemptive anthelmintische Therapie (z. B. mit Albendazol) wird nicht empfohlen. Aus Sicht der Autoren dieser Handlungsempfehlungen führt eine solche Therapie zu einer zu hohen Exposition von nichtinfizierten Kindern und Jugendlichen mit einem für diese Indikation nichtzugelassenen Medikament und damit verbundenen potenziellen Nebenwirkungen.

#### Syphilis (*Treponema pallidum*)

Aufgrund unzureichender Datenlage ist ein generelles Screening auf Syphilis bei minderjährigen Flüchtlingen nicht empfohlen.

Stattdessen sollten alle Schwangeren – wie in den Mutterschaftsrichtlinien vorgesehen – serologisch auf eine Lues untersucht werden.

#### Zystizerkose

Ein Screening auf Neurozystizerkose ist bei asymptomatischen minderjährigen Flüchtlingen nicht empfohlen.

Auswertungen des in Italien durchgeführten nationalen Screeningprogramms zeigten, dass symptomorientierte Untersuchungen bei Kindern mit anamnestischen Krampfanfällen oder neurologischer Symptomatik wahrscheinlich angemessener sind als eine Routineuntersuchung aller minderjähriger Flüchtlinge [21].

#### Zystische Echinokokkose (*Echinococcus granulosus*)

Ein Screening auf zystische Echinokokkose ist bei minderjährigen Flüchtlingen nicht empfohlen.

Es liegen keine ausreichenden Daten vor, die den Nutzen eines Screenings bei asymptomatischen Personen belegen.

## Impfungen

Untersuchungen haben gezeigt, dass je nach Herkunftsland bei der Mehrzahl der Flüchtlinge im Kindes- und Jugendalter ein positiver Antikörperstatus gegen impfpräventable Erkrankungen vorliegt (MMRV 70–95 % [22], Diphtherie und Tetanus 56–76 % [23]). Insbesondere bei jungen Kindern aus akuten Krisengebieten besteht das Risiko, dass die im Herkunftsland empfohlenen Impfungen nicht oder nur unvollständig durchgeführt wurden [24].

Daher sind die Überprüfung des Impfstatus und die Durchführung von Grundimmunisierung bzw. Nachhol- und Auffrischimpfungen wichtige Maßnahmen bei allen Flüchtlingen im Kindes- und Jugendalter, die frühzeitig nach Ankunft in Deutschland durchgeführt werden sollen.

Grundsätzlich sollten bei der Planung der Impfungen bisherige Impfdokumente berücksichtigt werden. Allerdings liegen diese Dokumente nur bei einem kleinen Teil der Flüchtlinge vor. Kinder- und Jugendliche ohne offizielle Impfdokumente gelten in der Regel als nicht oder unvollständig geimpft und sollten altersentsprechend alle Impfungen gemäß den aktuellen STIKO-Nachholimpfempfehlungen erhalten. Von Impftiter-Bestimmungen wird wegen deren Unzuverlässigkeit im Hinblick auf frühere Impfungen abgeraten. In Ausnahmefällen können glaubwürdige mündliche Aussagen zu vorangegangenen Impfungen berücksichtigt werden (z. B. syrische Kinder, die vor 2010 geboren sind, da die Impfquoten zu diesem Zeitpunkt in Syrien höher als in Deutschland lagen).

Wichtig ist eine einheitliche Dokumentation aller vorangegangenen sowie in Deutschland durchgeführten Impfungen. Sinnvoll ist es hierfür, bei der ersten Impfung den in Deutschland üblichen Impfpass anzulegen. Angaben aus offiziellen Impfdokumenten des Herkunftslandes und ggf. in der Impfplanung berücksichtigte mündliche Aussagen zu vorangegangenen Impfungen sollten bei dieser Gelegenheit in das neue Impfdokument übertragen werden.

Sinnvoll ist es, je nach Unterbringung, Alter der Flüchtlinge und ggf. auch aktuellem Ausbruchsgeschehen im Rahmen der Erstvorstellung eine Priorisierung der Impfungen durchzuführen. Im Rahmen nationaler Impfkampagnen, wie z. B. der COVID-19-Impfungen, sollte zudem sichergestellt werden, dass Flüchtlinge und Migranten ebenfalls einen direkten Zugang zu diesen Präventionsmaßnahmen haben.

Bei Unterkunft in Gemeinschaftseinrichtungen oder (anstehendem) Besuch von Kindergarten oder Schule ist folgende Priorisierung empfohlen:

### Standardimpfungen

- *Erste* Priorität: MMRV (Kinder ≥ 9 Monate),
- *nachfolgend:* DTaP-IPV-Hib-HBV (bei Kindern < 5 Jahre) bzw. Tdap-IPV (bei Kindern ≥ 5 Jahre) sowie Hepatitis B, PCV13 (bei Kindern < 2 Jahre), Men C (besser Men ACWY und Men B^2^), HPV (ab 9 Jahre), Rotavirus (Säuglinge < 6 Monate).

### Ausgewählte Indikationsimpfungen (grundsätzlich hohe Priorität)

- COVID-19 (altersabhängige Indikation),
- Influenza (saisonale Impfung),
- Hepatitis A (bei Ausbruchsgeschehen),
- FSME (bei Aufenthalt in Endemiegebieten [25]).

## Screening auf multiresistente Erreger

Vor einem stationären Aufenthalt ist bei minderjährigen Flüchtlingen ein Screening auf multiresistente Erreger (MRE) empfohlen, wenn der Patient

1. innerhalb der letzten 12 Monate im Herkunftsland bzw. im Transit einen Krankenhausaufenthalt oder wiederholten Kontakt mit Einrichtungen des Gesundheitssystem hatte
  oder
2. eine bekannte frühere Kolonisierung oder Infektion mit MRE hatte
  oder
3. chronische Wunden/Hautläsionen aufweist.

Darüber hinaus sollte ein MRE-Screening erfolgen, wenn der Patient in der Klinik Kontakte zu besonders vulnerablen Mitpatienten, z. B. aus den Bereichen Intensivmedizin, Kinderchirurgie, Onkologie oder Transplantationsmedizin, hat und

1. eine Flüchtlingsanamnese in den letzten 3 Monaten hat
  oder
2. in einer Gemeinschaftseinrichtung untergebracht ist.

Zum Screening benötigte Proben werden von folgenden Körperstellen abgenommen:

- beide Nasenvorhöfe und Rachen (MRSA) – 1 Tupfer,
- Abstrich von chronischen (schlecht heilenden) Wunden (MRSA),
- Rektalabstrich (multiresistente gramnegative Bakterien; MRGN) – ein Tupfer ausreichend, Durchtritt durch den Analsphinkter notwendig!
- Bei Kindern und Jugendlichen, die vor oder während ihrer Flucht in stationärer medizinischer Behandlung waren und die stationär im Krankenhaus aufgenommen werden müssen, soll zusätzlich ein Screening auf Carbapenem-resistente Acinetobacter spp. (auf entsprechenden Selektivmedien; mit dem Labor abzustimmen) erwogen werden. Dazu erfolgt ein Abstrich (Kombiabstrich) bukkal, von beiden Axillae, Leisten und rektal [26].

Bis zum Erhalt der MRE-Screening-Ergebnisse wird bei stationären Patienten eine Kontaktisolierung im Einzelzimmer empfohlen (soweit dies die baulichen Bedingungen bzw. die Aufnahmekapazitäten zulassen). Selbstverständlich können Kinder aus der gleichen Familie (bzw. der gleichen Wohnung) dabei kohortiert werden.

## Praktische Durchführung/Dokumentation

Flüchtlinge im Kindes- und Jugendalter werden nach ihrer Ankunft in Deutschland häufig zunächst für kurze Zeit in Erstaufnahmeeinrichtungen und nachfolgend in Gemeinschaftsunterkünften oder Wohnungen untergebracht. Hierdurch kommen die Kinder- und Jugendlichen häufig an verschiedenen Stellen mit dem Gesundheitswesen in Kontakt.

Frühzeitig nach Ankunft in Deutschland sollte eine Untersuchung aller minderjährigen Flüchtlinge durch einen Arzt, idealerweise für Kinder- und Jugendmedizin, erfolgen. Die Untersuchung sollte, wenn möglich in Anwesenheit der Eltern bzw. Sorgeberechtigen erfolgen. Zudem muss eine adäquate Verständigung sichergestellt werden, wozu häufig ein Dolmetscher notwendig ist.

Wichtig ist eine strukturierte Dokumentation der Untersuchungsergebnisse. Hierdurch werden ein Verlust relevanter Befunde und redundante Untersuchungen vermieden.

Für die Dokumentation von Anamnese, Untersuchungsbefunden sowie der Laborergebnisse steht z. B. das Bremer Gesundheitsheft zur Verfügung [2].

Impfungen sollten darüber hinaus im offiziellen gelben Impfausweis dokumentiert werden. Zudem sollen die Patienten aufgefordert werden, digitale Kopien der Dokumentation auf ihrem Mobiltelefon zu speichern.

## Checkliste Handlungsempfehlungen

(Tab. 4)

**Table 4:** 

Wer	Was
*Alle*	Anamnese zu aktuellen und chronischen Erkrankungen, inkl. Impfstatus und stattgehabte Operationen
	Familienanamnese und Begleitpersonen
	Anamnese zur Flucht
	Relevante psychische Erkrankungen oder Belastungen
	Anthropometrische Daten
	Allgemeine körperliche Untersuchung
	Weitere Untersuchungen: Seh- und Hörtest, Entwicklungsstatus
	Blutdruck
	Impfstatus (inkl. Nachholimpfungen und Dokumentation)
	Blutbild mit Differenzialblutbild
	Tuberkulose: IGRA oder ggf. THT (< 5 Jahre); ggf. ab 15 Jahre Thoraxröntgen
	Hepatitis B-Serologie (HBs-Antigen)
*Herkunft*	Hepatitis C-Serologie (HCV-Antikörper)
	HIV1/2-Screening-Test (p24-Antigen + HIV1/2-Antikörper)
	Schistosomiasis (Serologie)
	*Trypanosoma cruzi* (Serologie)
*Risiko*	Strongyloidose (Serologie): vor geplanter Immunsuppression
	MRE-Screening: vor stationärem Aufenthalt bei Risiko-Konstellation

Ein Screening ist empfohlen bei Herkunft aus einem Land mit erhöhter Prävalenz

*Hepatitis C:* Afrika, Naher und Mittlerer Osten, Osteuropa

*HIV:* Afrika südlich der Sahara, Osteuropa, zudem sollte ein Test bei Risikokonstellationen und in jeder Schwangerschaft erfolgen. Weiterhin ist es sinnvoll, generell im Rahmen der Erstuntersuchung einen Test anzubieten

*Schistosomiasis:* insbesondere Subsahara-Afrika und bei Herkunft oder Aufenthalt entlang des Nils

*Trypanosoma cruzi:* Lateinamerika (insbesondere Bolivien)

## Ausblick und politische Forderungen

Seit 2015 kamen jährlich zwischen 40.000 und 200.000 minderjährige Flüchtlinge nach Deutschland. Auch für die kommenden Jahre ist eine hohe Zahl an Flüchtlingen zu erwarten, aktuell insbesondere aus dem Kriegsgebiet in der Ukraine. Die medizinische Versorgung dieser Kinder und Jugendlichen ist von großer Bedeutung, in vielerlei Hinsicht jedoch herausfordernd. Die Sprachbarriere erfordert häufig den Einsatz eines im medizinisch-fachlichen Sinne vertrauenswürdigen Dolmetschers. Kulturelle Besonderheiten sind zu berücksichtigen. Der besonders wichtige Besuch von Kindergarten, Schulen und Sportvereinen etc. (Integration und Teilhabe, schneller Erwerb u. a. von Sprachkenntnissen) soll organisiert und vermittelt werden. Medizinische Befunde aus dem Herkunftsland sind häufig nicht vorhanden, und die Durchführung des in dieser Leitlinie vorgeschlagenen Untersuchungsprogramms erfordert spezielle medizinische Kenntnisse sowie personelle und räumliche Ressourcen.

Für all diese Dinge gibt es aber weder im stationären noch im ambulanten Sektor ausreichende personelle Ressourcen oder eine adäquate Vergütung. Wünschenswert ist es daher, dass für die auch in Zukunft zu erwartende hohe Zahl minderjähriger Flüchtlinge die Rahmenbedingungen für eine adäquate medizinische Versorgung mit strukturierten Erst- und Vorsorgeuntersuchungen entwickelt werden.

## Hilfreiches Material und Links

1. Informationen zu Impfungen des Robert Koch-Instituts, jährlich publiziert in *Epidemiologisches Bulletin* 4, zuletzt: https://www.rki.de/DE/Content/Infekt/EpidBull/Archiv/2022/Ausgaben/04_22.pdf?__blob=publicationFile
2. Übersicht über die in einzelnen Ländern empfohlenen Impfungen
  ECDC: https://vaccine-schedule.ecdc.europa.eu/
  sowie
  WHO: https://apps.who.int/immunization_monitoring/globalsummary/schedules
3. Internationale Perzentilen
  WHO child growths standards based on length/height, weight and age: https://www.who.int/tools/child-growth-standards [16]
4. Bremer Gesundheitsheft
https://www.gesundheitsamt.bremen.de/fluechtlinge-15222?wdLOR=c09AA024E-4241-0846-AC6D-B6C95465D9C0&web=1
5. Medizinische Maßnahmen bei immigrierenden Kindern und Jugendlichen – Aktualisierung vom 28.02.2018 (Empfehlungen der Kommission für Infektionskrankheiten und Impffragen sowie der Kommission für Globale Kindergesundheit der Deutschen Akademie für Kinder- und Jugendmedizin [DAKJ])
https://www.dakj.de/stellungnahmen/medizinische-massnahmen-bei-immigrierenden-kindern-und-jugendlichen-langversion/
6. Stellungnahme der Deutschen Akademie für Kinder- und Jugendmedizin (DAKJ) zur Situation von unbegleiteten minderjährigen Flüchtlingen
https://www.dakj.de/stellungnahmen/stellungnahme-der-deutschen-akademie-fuer-kinder-und-jugendmedizin-dakj-zur-situation-von-unbegleiteten-minderjaehrigen-fluechtlingen/
7. Stellungnahme der Kommission Globale Kindergesundheit der Deutschen Akademie für Kinder- und Jugendmedizin (DAKJ) zur pädiatrischen Gesundheitsversorgung von minderjährigen Flüchtlingen und Asylbewerbern
https://www.dakj.de/stellungnahmen/paediatrische-gesundheitsversorgung-von-minderjaehrigen-fluechtlingen-und-asylbewerbern/
8. Hinweise des Robert Koch-Instituts für den Öffentlichen Gesundheitsdienst (ÖGD) und die Ärzteschaft zum Management von Ausbrüchen in Gemeinschaftsunterkünften von Asylsuchenden
https://www.rki.de/DE/Content/GesundAZ/A/Asylsuchende/Management_Ausbrueche.html
9. Stellungnahme des Robert Koch-Instituts zur Untersuchung von Tuberkulose bei asylsuchenden Kindern und Jugendlichen unter 15 Jahren
https://www.rki.de/DE/Content/InfAZ/T/Tuberkulose/Tuberkulose-Screening_Kinder.html
10. Impfaufklärungsvideo in verschiedenen Sprachen: https://www.refudocs.de/startseite/
11. Erklärung wichtiger Aspekte der Tuberkulose: https://www.explaintb.org/app-3/
12. Überblick über BCG-Impfschemata in einzelnen Ländern: http://www.bcgatlas.org

## Begründung der vorgeschlagenen Screeninguntersuchungen

### Blutbild

Untersuchungen bei Flüchtlingen im Kindes- und Jugendalter in Deutschland [27] und Kanada [28] ergaben übereinstimmend eine Prävalenz von rund 22 % für Anämien (Hämoglobin unterhalb des altersentsprechenden Referenzwertes). Das höchste Risiko einer Anämie besteht bei Kindern unter 5 Jahren sowie schwangeren Frauen [29]. Ursächlich für das erhöhte Risiko einer Anämie bei Flüchtlingen sind in erster Linie ein unzureichender Ernährungsstatus (Eisenmangel) sowie Hämoglobinopathien (u. a. Thalassämie, Sichelzellerkrankung), Infektionserkrankungen (z. B. intestinale Helminthosen, Schistosomiasis) und andere chronische Erkrankungen [30]. Es ist bekannt, dass Anämien bei schwangeren Frauen einen Risikofaktor für Frühgeburtlichkeit und niedrigeres Geburtsgewicht darstellen [31]. Darüber hinaus werden bei Kindern negative Auswirkungen auf die neurologische Entwicklung diskutiert [32].

Anämien sind durch ein Blutbild einfach und sicher zu diagnostizieren und in vielen Fällen nachfolgend auch einfach behandelbar. Daher wird die Bestimmung eines Blutbildes nach Ankunft für alle Flüchtlinge im Kindes- und Jugendalter empfohlen.

Eine Eosinophilie (> 500–1000/µl; Grenzwert in der Literatur nicht eindeutig definiert) im peripheren Blut sollte zum Anlass genommen werden, die Diagnostik zum Nachweis oder Ausschluss einer Filariose, einer Infektion mit Schistosomen, Zwergfadenwurm (*Strongyloides* spp.), Zwergbandwurm (*Hymenolepis* spp.) und/oder intestinalen Helminthen zu erwägen und ggf. mit dem parasitologischen Labor abzusprechen. Anzumerken ist hier, dass die Datenlage zu dem diagnostischen Wert der Eosinophilie bei minderjährigen Flüchtlingen begrenzt ist. Insgesamt scheint die Spezifität und Sensitivität der Eosinophilie hinsichtlich parasitärer Erkrankungen begrenzt zu sein [20, 33]. Eine Stuhldiagnostik auf intestinale Parasitosen sollte nicht routinemäßig, sondern nur bei klinischem Verdacht durchgeführt werden.

Eine Diagnostik auf Hämoparasitosen, wie Malaria oder Leishmaniose, wird ebenfalls nur bei Auftreten von Symptomen empfohlen.

### Tuberkulose

Begründet wird die medizinische Notwendigkeit eines routinemäßigen Screenings mit der erhöhten Prävalenz der Tuberkulose innerhalb von Risikopopulationen wie Flüchtlingen und Migranten speziell aus Hochendemiegebieten.

Bei in den vergangenen Jahren in Deutschland durchgeführten Untersuchungen lag die Rate positiver IGRA-Testergebnisse unter minderjährigen Flüchtlingen in Deutschland im Bereich 6,8 % (Kinder bis 15 Jahre) [34] bis 13,9 % (unbegleitete minderjährige Flüchtlinge) [35].

Zum Vergleich beträgt die Tuberkulose-Inzidenz in der deutschen Allgemeinbevölkerung ca. 6 pro 100.000.

Interessanterweise zeigten die in Deutschland erfolgten Untersuchungen eine vergleichbare Prävalenz der latenten Tuberkulose bei Kindern aus bekannten Hoch- (z. B. Afghanistan) und Niedrigprävalenzländern (z. B. Syrien) [34]. Hier kann es im Rahmen der Flucht und unter erschwerten Lebensbedingungen mit reduzierter Hygiene zu einer Tuberkulose-Exposition gekommen sein.

In einem Review, der die nationalen Prävalenzdaten mit jenen in Krisenregionen verglich, konnte zudem gezeigt werden, dass die Raten in Krisenregionen 2‑ bis 20-fach erhöht sind [36].

Aufgrund dieser Erkenntnisse erscheint es sinnvoll und pragmatisch, bei der Durchführung eines Tuberkulosescreenings keinen Unterschied zwischen Kindern und Jugendlichen aus Herkunftsländern mit hoher und niedriger Tuberkuloseprävalenz zu machen.

In die Abwägung eines routinemäßigen Tuberkulosescreenings muss miteinbezogen werden, dass das Risiko einer Tuberkulose-Übertragung durch Kinder deutlich geringer ist als durch Erwachsene (auch wenn Ansteckungen durch Sputum-negative Kinder und durch Sauglinge in Einzelfällen beschrieben sind). Andererseits ist das Risiko, bei einer Tuberkulose-Infektion eine aktive Tuberkulose zu entwickeln, in den ersten Lebensjahren deutlich erhöht. Insbesondere bei jungen Kindern kann das Erkennen einer latenten Tuberkulose mit nachfolgender Chemoprävention somit schwere Krankheitsverläufe verhindern. Schließlich haben Kinder und Jugendliche aufgrund ihres noch jungen Alters eine längere Lebensspanne, in der sich eine latente Tuberkulose zu einer aktiven Erkrankung entwickeln kann. Das Screening auf eine Tuberkulose kann somit die Erkrankung beim individuellen Patienten verhindern und gleichzeitig die Anzahl infektiöser Patienten in der Zukunft reduzieren. Wenn möglich sollten bei einem bekannten Expositionsereignis im Umfeld des Kindes die mikrobiologischen Informationen zum Index-Fall erhoben werden. Insbesondere bei Kindern aus Gebieten mit einer höheren MDR-TB-Rate (z. B. der Ukraine) kann nur so eine adäquate Chemoprävention sichergestellt werden.

Angesichts des aktuellen Krieges in der Ukraine ist anzumerken, dass dort die Tuberkulose-Inzidenz mit 73 Fällen pro 100.000 Einwohnern hoch ist. In der Ukraine treten auch häufig multiresistente Tuberkulose-Fälle (MDR-TB) auf (24–29 % bei Neudiagnosen bzw. 32–46 % bei vorbehandelten Personen) [37]. Daher zählt die WHO die Ukraine zu den High Burden Countries für MDR-TB [38]. Ebenso stellen Koinfektionen mit HIV ein relevantes Problem dar. Etwa 260.000 Menschen sind in der Ukraine mit HIV infiziert, was rund 0,6 % der Gesamtbevölkerung entspricht. In > 20 % der Fälle liegt bei einer Tuberkulose gleichzeitig eine HIV-Infektion vor [37]. Grundsätzlich gilt es, eine rasche Weiterbehandlung von bereits diagnostizierten und therapierten Kindern und Jugendlichen sowie Erwachsenen zu ermöglichen, da eine Unterbrechung der Behandlung den Therapieerfolg gefährdet und zu weiteren Medikamentenresistenzen führen kann.

Aktuelle Tuberkulose-Inzidenzen werden jährlich im Tuberkulose Report der WHO publiziert [38] und sind auch als App verfügbar [39].

Detaillierte Informationen zur Testung sowie zur Therapie der Tuberkulose im Kindes- und Jugendalter finden sich in der AWMF-S2k-Leitlinie zu Diagnostik, Prävention und Therapie der Tuberkulose im Kindes- und Jugendalter [17].

### Hepatitis B

Eine chronische Hepatitis B (HBs-Ag-Positivität) ließ sich in verschiedenen Untersuchungen bei 3,3 % (Erwachsene ab 15 Jahre [40]) bzw. 7,7 % (unbegleitete minderjährige Flüchtlinge [20]) nachweisen. Die Prävalenz eines positiven HBs-Ag-Nachweises ist dabei stark herkunftsabhängig und liegt bei Flüchtlingen aus Afrika südlich der Sahara häufig bei > 10 %, während bei Flüchtlingen aus Irak, Syrien und Afghanistan Prävalenzen zwischen etwa 1 und 4 % berichtet wurden [40]. Zum Vergleich liegt die HBs-Ag-Prävalenz in der deutschen Allgemeinbevölkerung bei 0,4 % [41], bei Kindern ist eine noch niedrigere Prävalenz zu erwarten.

Insgesamt besteht somit bei allen derzeitigen Flüchtlingsgruppen eine im Vergleich zur deutschen Allgemeinbevölkerung erhöhte HBs-Ag-Prävalenz. Bei Kindern und Jugendlichen mit chronischer Hepatitis-B-Infektion besteht eine Indikation zu regelmäßiger Überwachung der Infektiosität und Leberfunktion. In bestimmten Fällen ist eine antivirale Therapie angezeigt. Als weitere Konsequenz wird der Hepatitis-B-Impfstatus bei engen Kontaktpersonen serologisch überprüft und gegebenenfalls komplettiert. Eine Metaanalyse ergab, dass ein HBV-Screening bei Erwachsenen und Kindern aus Ländern mit erhöhter HBV-Prävalenz mit anschließender Behandlung und präventiver Impfung enger Kontaktpersonen kosteneffektiv ist [42].

Aufgrund der je nach Alter und Herkunftsland gering bis stark erhöhten Wahrscheinlichkeit einer chronischen Hepatitis B-Infektion, des einfachen serologischen Nachweises mittels HBs-Ag sowie der daraus resultierenden therapeutischen Konsequenz wird die Bestimmung des HBs-Ag für alle Flüchtlinge im Kindes- und Jugendalter empfohlen.

Anzumerken ist, dass die Wahrscheinlichkeit einer Hepatitis-B-Infektion für bestimmte Flüchtlingsgruppen (vor allem junge Kinder aus Syrien und dem Irak) vermutlich gegenüber der deutschen Allgemeinbevölkerung nur leicht erhöht ist [43]. Allerdings liegen hierzu nur beschränkt Daten vor, und es wird auch in Zukunft kaum möglich sein, für alle Altersgruppen, Herkunftsländer und Fluchtwege konkrete und aktuelle Daten zur HBs-Ag-Prävalenz zu erhalten. Aus pragmatischen Gründen scheint somit eine generelle Screeningempfehlung gerechtfertigt.

### Hepatitis C

Die Prävalenz eines positiven Hepatitis-C-Antikörper (Anti-HCV)-Nachweises lag in einer deutschen Untersuchung bei Flüchtlingen und Asylsuchenden über alle Altersstufen bei 1,2 %, bei insgesamt 0,7 % war HCV-RNA und somit eine chronische Hepatitis C nachweisbar [44]. In einer Metaanalyse lag die Anti-HCV-Prävalenz über alle Altersstufen bei 1,9 %, bei Kindern bei 0,6 % [45]. Die Prävalenz der Hepatitis C steigt somit mit zunehmendem Alter an und ist zudem stark herkunftsabhängig, mit den höchsten Infektionsraten in Ländern Afrikas und Asiens [45]. Die Prävalenz der Hepatitis-C-Infektion lag bei vorangegangenen Untersuchungen in der deutschen Allgemeinbevölkerung bei 0,3 % [46] und somit unterhalb der Prävalenz in den verschiedenen Flüchtlingsgruppen.

Kinder mit einer chronischen Hepatitis C sind häufig für lange Zeit asymptomatisch. Nach perinataler Infektion besteht die Chance einer Spontanheilung, bei Kindern > 3 Jahre wird diese jedoch unwahrscheinlich [47]. In den letzten Jahren gab es wesentliche Fortschritte in der antiviralen Therapie der Hepatitis C mit vom Serotyp-abhängigen Heilungsraten von > 95 %. Bei Kindern besteht eine Zulassung für antivirale Medikamente ab dem Alter von 3 Jahren (Stand März 2022). Daher wird empfohlen, die Bestimmung von Anti-HCV insbesondere Flüchtlingen aus Hochprävalenzländern anzubieten. Hierzu gehören beispielsweise zahlreiche afrikanische Länder sowie weite Gebiete des Nahen und Mittleren Ostens (unter anderem Ägypten, aber auch Syrien und Pakistan).

### HIV

Die Seroprävalenz von HIV-Antikörpern lag in deutschen Untersuchungen bei erwachsenen Flüchtlingen bei 0,25 % [48] bzw. 0,4 % bei unbegleiteten minderjährigen Flüchtlingen [20]. Die größte Untersuchung mit mehr als 95.000 Personen wurde an erwachsenen Flüchtlingen durchgeführt. Hier war der Antikörper-Suchtest bei 0,7 % positiv, nachfolgend wurde bei 0,3 % eine HIV-Infektion diagnostiziert. Insgesamt entsprach die HIV-Prävalenz bei den untersuchten Flüchtlingen weitgehend der bekannten Prävalenz der Herkunftsländer. So wurde bei Flüchtlingen aus Ländern mit niedriger HIV-Prävalenz in 0,03 % (Syrien) bzw. 0,05 % (Afghanistan) eine HIV-Infektion festgestellt, während die Prävalenz bei Herkunft aus Afrika südlich der Sahara deutlich höher lag [40]. Ein erhöhtes Infektionsrisiko besteht zudem bei Flüchtlingen, insbesondere jungen Frauen, mit Sekundärmigration, also nach einem Aufenthalt in einem Transitland [49].

Zum Vergleich liegt die HIV-Prävalenz in der deutschen Allgemeinbevölkerung bei etwa 0,1 %.

Aktuell sind mehr als die Hälfte aller HIV-positiven Minderjährigen in Europa Migranten. In dieser Gruppe liegt der Beginn einer antiretroviralen Therapie in einem späteren Lebensalter, und es zeigt sich ein Trend zu höherer Progression zu AIDS und Todesfällen im Vergleich zu in Europa geborenen Kindern- und Jugendlichen [50].

Zusammenfassend besteht für minderjährige Flüchtlinge aus Hochprävalenzländern (insbesondere Subsahara-Afrika, aber auch Osteuropa) sowie für bestimmte Risikogruppen eine erhöhte Wahrscheinlichkeit einer HIV-Infektion und ein besonderes Risiko für eine zu späte Therapie der Erkrankung. Die Gelegenheit einer Untersuchung nach Ankunft in Deutschland sollte zum Anlass genommen werden, allen minderjährigen Flüchtlingen, insbesondere bei Herkunft aus Hochprävalenz-Ländern, ein HIV-Screening anzubieten.

### Schistosomiasis

Weltweit ist die Schistosomiasis eine häufige Erkrankung. Es wird geschätzt, dass mehr als 200 Mio. Menschen infiziert sind. Über 90 % der Infektionen treten in Afrika südlich der Sahara bzw. entlang des Nils auf. Neben Afrika ist die intestinale Schistosomiasis auch in Teilen Asiens und Südamerikas vertreten (unter anderem östliches Brasilien, Venezuela, Jemen, China, Laos, Kambodscha, Philippinen, Sulawesi in Indonesien). Die urogenitale Form der Schistosomiasis (*Schistosoma haematobium*) ist mit minimalen Ausnahmen ausschließlich auf den afrikanischen Kontinent begrenzt.

Bei einer Untersuchung an 164 unbegleiteten minderjährigen Flüchtlingen (hiervon > 90 % aus Afrika südlich der Sahara) wurden eine intestinale Schistosomiasis bei rund 7 % und eine urogenitale Schistosomiasis bei rund 6 % in Stuhl- bzw. Urinproben nachgewiesen [20]. Die Rate einer positiven Schistosomiasis-Serologie als Hinweis auf eine chronische oder bereits ausgeheilte vorangegangene Infektion lag in einer deutschen Untersuchung bei minderjährigen Flüchtlingen aus Afrika südlich der Sahara bei rund 25 % [51], in einer Untersuchung aus Italien bei > 90 % [52].

Die Schistosomiasis kann oral mit Praziquantel einfach und sicher behandelt werden. Unbehandelt besteht die Gefahr schwerwiegender Organkomplikationen. Daher wird ein Screening aller Flüchtlinge aus Ländern Afrikas südlich der Sahara oder entlang des Nils bzw. mit Herkunft aus Endemiegebieten mittels einer Schistosomiasis-Serologie empfohlen. Der Nachweis spezifischer Antikörper ist dann entsprechend der aktuellen AWMF-Leitlinie abzuklären [19].

### Strongyloidose

Die Strongyloidose ist eine weltweit häufige Parasitose, die vor allem in tropischen und subtropischen Gebieten auftritt. Die Infektion kann durch Autoinfektion über Jahrzehnte überwiegend asymptomatisch persistieren. Bei Immunsuppression kann sich jedoch ein lebensbedrohliches Krankheitsbild entwickeln [53, 54].

Bei 119 Stuhluntersuchungen von unbegleiteten minderjährigen Flüchtlingen wurde in 0,8 % der Fälle eine Strongyloidose festgestellt [20]. In einer Metaanalyse wurde die Rate positiver Serologie-Befunde mit rund 10 % aller Flüchtlinge angegeben, mit der höchsten Rate (16,8 %) bei Herkunft aus Ländern Afrikas südlich der Sahara [43].

Die Strongyloidose wird mit Ivermectin behandelt, wodurch bei erstmaliger Therapie Heilungsraten von rund 80 % erzielt werden [55].

Da die chronische Infektion bei Menschen ohne schwere Vorerkrankungen fast immer asymptomatisch bleibt, erscheint ein generelles Screening aller Flüchtlinge nicht gerechtfertigt. Bei Risikopersonen, insbesondere bei Vorliegen einer HIV-Erkrankung oder bei bzw. vor geplanter Immunsuppression sollte eine gezielte Untersuchung mittels Serologie sowie spezieller parasitologischer und molekularbiologischer Stuhldiagnostik durchgeführt werden.

### Venerische Syphilis (*Treponema pallidum*)

Bei Kindern ist insbesondere die konnatale Syphilis von klinischer Bedeutung. Es wird geschätzt, dass jährlich mehr als eine Million Fälle konnataler Syphilis auftreten [56]. Eine vertikal übertragene Syphilis kann zu Aborten und Frühgeburtlichkeit führen. Bei infizierten Neugeborenen besteht ein weites Spektrum klinischer Symptome, nicht selten sind infizierte Neugeborene aber asymptomatisch. Auch bei primär asymptomatischen Neugeborenen kann sich im Verlauf eine konnatale Syphilis spät klinisch manifestieren. Diese Lues connata tarda ist definiert durch das Auftreten klinischer Symptome im Alter > 2 Jahre und manifestiert sich an einer Vielzahl von Organen, u. a. als Keratitis und sensorineurale Schwerhörigkeit.

Durch eine Behandlung der Schwangeren sowie durch eine frühzeitige Behandlung der infizierten Säuglinge in den ersten 3 Lebensmonaten kann die klinische Manifestation der Lues connata meist erfolgreich verhindert werden.

Die Seroprävalenz von *Treponema-pallidum*-Antikörpern lag bei Untersuchungen an Flüchtlingen in Deutschland bei 0,13 % (alle Altersgruppen [48]). Dabei kann der Screeningtest nicht zwischen venerischen und nichtvenerischen Treponematosen (z. B. Frambösie, Pinta) unterscheiden. Deswegen erfordert ein positiver Screeningtest eine weitere Abklärung. Aktuelle Daten zur Prävalenz der Syphilis bei minderjährigen Flüchtlingen liegen nicht vor.

Aufgrund dieses Mangels an Daten und der eingeschränkten Therapieeffektivität bei Kindern älter als 3 Monaten wird kein generelles Screening auf *Treponema pallidum* empfohlen. Stattdessen soll entsprechend den Mutterschaftsrichtlinien eine gezielte Untersuchung von Schwangeren durchgeführt werden. Zudem sollten behandelnde Ärzte die Möglichkeit einer konnatalen Syphilis bei minderjährigen Flüchtlingen bei entsprechenden Symptomen bedenken.

### Chagas-Krankheit (*Trypanosoma cruzi*)

Die Chagas-Krankheit ist in Lateinamerika endemisch, die Verbreitung reicht vom Süden der USA bis in den Norden von Argentinien und Chile [57]. Die höchsten Erkrankungsraten werden aus Bolivien berichtet, hier kann eine Infektion mit *T. cruzi* bei etwa 15 % der schwangeren Frauen [58] und etwa 60 % der Personen mit Herzerkrankungen nachgewiesen werden [59].

Bei Kindern ist besonders die konnatale Chagas-Erkrankung relevant. Etwa 1–10 % der Neugeborenen von infizierten schwangeren Frauen entwickeln eine konnatale Infektion. Als klinische Manifestation treten ein niedriges Geburtsgewicht, Anämien sowie selten schwere neurologische und respiratorische Komplikationen auf [60].

Ein Screening von Migranten aus Lateinamerika wird in Spanien durchgeführt. Hierbei zeigte sich eine *T.-cruzi*-Prävalenz von rund 6 % [61]. Neugeborene infizierter Mütter werden auf das Vorliegen einer konnatalen Infektion untersucht. Im Falle einer konnatalen Infektion besteht eine Behandlungsindikation mit Benznidazol oder Nifurtimox [62]. Die Therapie ist gut verträglich und effektiv mit serologischen Heilungsraten von > 90 % [62, 63]. Eine ökonomische Analyse aus den USA zeigte, dass ein generelles maternales Screening bereits bei sehr geringer mütterlicher Prävalenz und niedrigen vertikalen Transmissionsraten eine kostensparende Maßnahme darstellt [64]. Aus diesen Gründen erscheint ein generelles Screening von Mädchen und Frauen im gebärfähigen Alter aus Lateinamerika (insbesondere Bolivien) sinnvoll.

Die chronische Chagas-Erkrankung manifestiert sich klinisch üblicherweise Jahre bis Jahrzehnte nach der Infektion durch eine Kardiomyopathie, seltener durch gastrointestinale Erkrankungen (z. B. Achalasie). Die Frage, ob eine frühe Behandlung einer Infektion die spätere Entwicklung der chronischen Chagas-Erkrankung verhindern kann, ist aufgrund der langen Zeit zwischen Erstinfektion und der Manifestation chronischer Erkrankung schwer zu beantworten. Generell wird jedoch davon ausgegangen, dass eine Behandlung möglichst frühzeitig nach der Primärinfektion erfolgen sollte.

Kinder im Alter von 7 bis 12 Jahren verlieren nach Behandlung mit Benznidazol zu mehr als 50 % Antikörper gegen *T. cruzi*, jedoch nur 5 % in der Placebo-Gruppe [65]. Aus diesem Grund wird eine generelle Screeningempfehlung für Kinder und Jugendliche aus Endemiegebieten ausgesprochen.

### Zystizerkose

Die Zystizerkose, die durch Larven des Schweinebandwurms (*Taenia solium*) verursacht wird, ist in vielen Gebieten Asiens, Afrikas südlich der Sahara sowie in Zentral- und Südamerika endemisch [21]. Hohe Prävalenzen intrazerebraler Verkalkungen als Zeichen einer Neurozystizerkose finden sich vor allem in ländlichen Gebieten [66]. Dort ist die Neurozystizerkose auch eine häufige Ursache von Epilepsie.

Allerdings entwickelt vermutlich nur ein kleiner Teil der infizierten Personen neurologische Symptome [67]. Zudem gibt es, bei dünner Datenlage, keinen Nachweis für einen positiven Effekt eines generellen serologischen Screenings bei asymptomatischen Personen [68]. Daher ist ein generelles Screening derzeit nicht empfohlen. Sinnvoll ist eine gezielte Diagnostik bei entsprechenden neurologischen Symptomen.

### Zystische Echinokokkose (*Echinococcus granulosus*)

Die zystische Echinokokkose ist endemisch unter anderem im Nahen Osten, Gebieten in Afrika südlich der Sahara sowie in Südamerika.

Infektionen des Menschen entstehen durch die Ingestion infektiöser Eier, die üblicherweise von Hunden ausgeschieden werden. Nach der Infektion entwickeln sich über einen Zeitraum von üblicherweise Jahren Zysten in verschiedenen Geweben. Betroffen sind hierbei am häufigsten die Leber sowie die Lunge.

Die Infektion ist zunächst asymptomatisch, bei Infektion im Kinder- und Jugendalter treten klinische Symptome zumeist erst im Erwachsenenalter auf.

Screeningmethode der Wahl zur Detektion der zystischen Echinokokkose ist eine Sonographie des Abdomens, die serologische Untersuchung weist im Vergleich eine niedrige Sensitivität auf [69, 70].

Allerdings liegen keine belastbaren Daten zur Prävalenz der zystischen Echinokokkose bei minderjährigen Flüchtlingen vor. Zudem erscheint ein generelles Screening mittels Sonographie wenig praktikabel. Daher kann derzeit kein generelles Screening empfohlen werden.

### Screening auf multiresistente Erreger (MRE)

Screeninguntersuchungen auf multiresistente Erreger (MRE) umfassen Methicillin-resistente *Staphylococcus aureus* (MRSA) sowie multiresistente gramnegative Erreger (MRGN). Innerhalb Europas nimmt die MRE-Prävalenz tendenziell von Norden nach Süden zu [71]. Dadurch ist auch bei Flüchtlingen, die aus Ländern mit erhöhter MRE-Prävalenz nach Deutschland kommen, mit einer vermehrten Kolonisation durch MRE zu rechnen. Besondere Risikofaktoren sind vorangegangene Krankenhausaufenthalte bzw. Kontakt zum Gesundheitssystem in Hochprävalenz-Ländern.

Zur MRE-Prävalenz bei minderjährigen Flüchtlingen liegen mehrere Untersuchungen bei stationär behandelten Patienten in Deutschland vor. Hierbei wurden MRE-Prävalenzen von rund 33 % [72] sowie in einer anderen Untersuchung eine MRGN-Prävalenz von rund 41 % festgestellt [73]. Die Prävalenz von MRE liegt bei minderjährigen Flüchtlingen somit deutlich höher als in der deutschen Allgemeinbevölkerung [73]. Weitere europäische Studien an Flüchtlingen sowohl im Kindes- [74] als auch im Erwachsenenalter [75] bestätigen die erhöhte Prävalenz von MRE. Mit zunehmender Dauer des Aufenthaltes in Deutschland ist eine Abnahme der MRE Prävalenz zu beobachten [73]. In einer aktuell zirkulierenden Information der Bundeswehr, erstellt in Zusammenarbeit mit dem Nationalen Referenzzentrum für gramnegative Krankenhauserreger (persönliche Kommunikation R. Bialek), wird die Häufigkeit von Resistenzen gegen Carbapeneme (4MRGN) bei Bakterienisolaten von Patienten in Gesundheitseinrichtungen in der Ukraine im Jahre 2020 mitgeteilt. Danach wiesen 5 % der *E.‑coli*-Isolate, 54 % der *Klebsiella pneumoniae* und 77 % der Isolate von *Acinetobacter* spp. eine Resistenz gegen Carbapeneme auf. Der optimale Ort zum Screening auf Bakterien der Gattung *Acinetobacter* wurde bislang nicht ermittelt. Studien von Ausbruchsgeschehen zeigen jedoch, dass die Nachweisrate Infizierter durch Untersuchungen von Hautabstrichen signifikant erhöht werden kann. In Anbetracht der o. a. Häufigkeit von 4MRGN *Acinetobacter* spp. wurde das MRE-Screening um einen kombinierten Abstrich der Innenseite der Wangen, der Haut beider Axillae und beider Leisten sowie des Rektums erweitert. Da die Abstriche im Labor auf MRGN unabhängig von der Spezies untersucht werden, beinhaltet dieser kombinierte Abstrich das MRE-Screening mittels isoliert entnommenen Rektalabstrich, der damit entfällt.

## Footnotes

1 Da die Medizin insgesamt und insbesondere die Pädiatrie ein überwiegend weibliches Fach ist, wäre es angemessen, alle Berufsbezeichnungen in der weiblichen Form zu führen. Dennoch haben sich die Autorinnen und Autoren letztlich im Sinne der einfacheren (weil gewohnten) Lesbarkeit für die männliche Form entschieden. Ausdrücklich soll dies alle Geschlechter (m/w/d) einbeziehen.

2 Diese beiden Impfungen sind bisher von der STIKO nur als Indikationsimpfungen geführt.
